# Carpal tunnel syndrome in children: a case report

**DOI:** 10.11604/pamj.2022.41.116.33182

**Published:** 2022-02-10

**Authors:** Sbai Mohamed Ali, Ben Omrane Youssef, Bellila Senda, Ouni Asma, Maalla Riadh

**Affiliations:** 1 Department of Plastic Surgery, Faculty of Medicine of Tunis, University El Manar Tunis, La Rabta, Tunis, Tunisia

**Keywords:** Carpal tunnel syndrome, child, mucopolysaccharidosis, hand surgery, case report

## Abstract

Carpal tunnel syndrome is the most common peripheral neuropathy in adults but remains a very rare condition in children. Lysosomal overload diseases (mucopolysaccharidosis and mucolipidosis), anatomical abnormalities, trauma and familial forms are the most frequent aetiologies. No studies report idiopathic forms. We present the case of a 14-year-old girl with Mucopolysaccharidosis type I (MPS I) in its moderate form with multi-visceral involvement. She had a history of a right sunken thumb operated on at a young age. Presenting with bilateral carpal tunnel syndrome more predominant and deficient on the right, confirmed by ultrasound and electromyography (EMG). The patient under went median nerve neurosis with a conventional approach. No complications were observed, we obtained a sensitive recovery at S4, on the motor level the patient is improving and at 6 months follow-up we obtained a motor score of M3. Carpal tunnel syndrome in children is rare. The clinical picture is not very noisy. It is a serious pathology because it can lead to significant motor sequela. It is therefore necessary to be vigilant and to have easy recourse to complementary examinations (electromyography, ultrasound). For our team, the therapeutic attitude is that of systematic open surgical treatment.

## Introduction

Mucopolysaccharidosis type I (MPSI) is a lysosomal overload disease related to the deficiency of a glycosaminoglycan (GAG) metabolism enzyme, alpha-L-Iduronidase (IDUA). It is a rare and severe recessive disease with a heterogeneous clinical spectrum including progressive visceral and bone involvement. In the hand the clinical manifestations are rare, represented by carpal tunnel syndrome and tenosynovitis of the flexors of the fingers related to infiltration of the retinaculum of the flexors of the synovial sheaths of the fingers. We present a case of a 14-year-old girl with MPSI, who had a carpal tunnel syndrome that progressed well after surgical treatment. The aim of this paper is to review the epidemiological, pathophysiological, clinical and therapeutic aspects of this rare condition.

## Patient and observation

**Figure 1:**
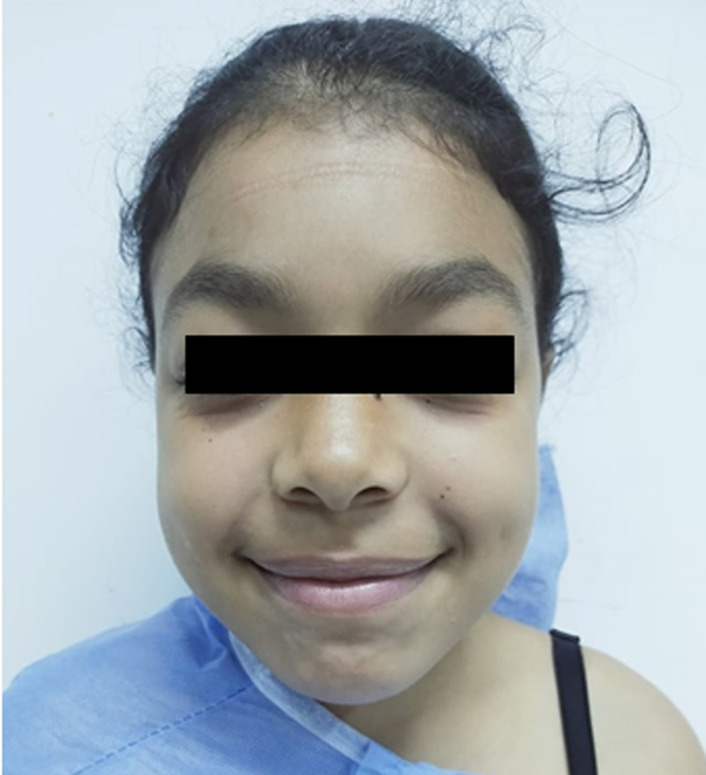
facial dysmorphia with thickened features

**Patient information:** a 14-year-old female patient from a non-consanguineous marriage was followed in pediatrics for a moderate form of MPS type 1 with multi-visceral involvement: facial dysmorphia (Figure 1), ear, nose, and throat (ENT) involvement, hepatomegaly, ophthalmic involvement, pulmonary involvement and joint involvement with bilateral genu valgum. She had a history of surgically operated right sore thumb. She presented with stiffness and paresthesia of all the fingers of both hands, more accentuated on the right side.

**Figure 2:**
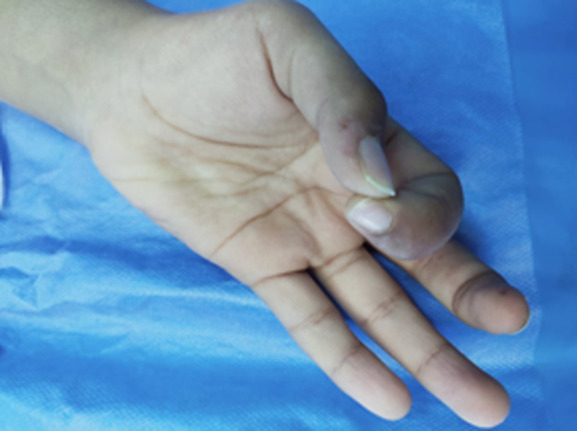
deficit of digital pollicis pincer

**Figure 3:**
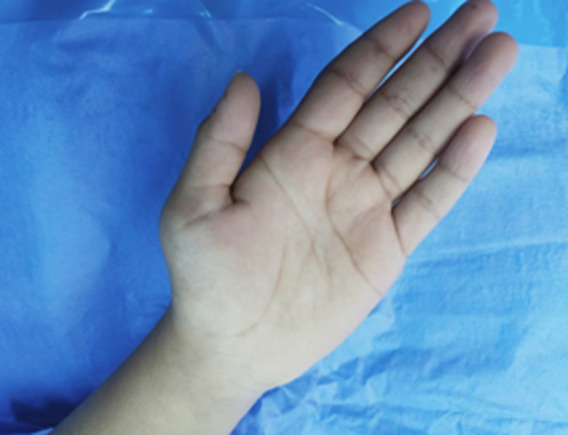
amyotrophy in thenar muscles

**Clinical findings:** the clinical examination found amyotrophic of the thenar muscles with a deficit of digital pollicis pincer (Figure 2, Figure 3), a positive Phalen's test and a pseudo-tinel on percussion of the median nerve at the entrance to the carpal tunnel.

**Diagnostic assessment:** electromyography examination noted a slowing of conduction of the median nerve and ultrasound showed thickening of the flexor retinaculum and a thinned appearance of the nerve within the carpal tunnel.

**Figure 4:**
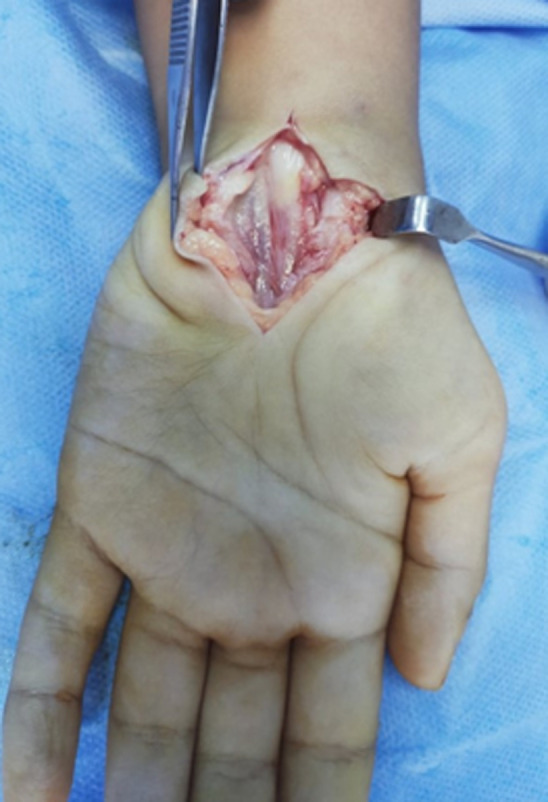
intra-operative photo showing the reduced bluish aspect of the medial nerve

**Therapeutic intervention:** we performed neurolysis of the right median nerve at the wrist under general anesthesia using the conventional approach, the exploration found a very thickened ligament, the nerve showed a neuroma at the entrance to the carpal tunnel of 8mm in diameter and a shrunken, bluish appearance of the nerve inside the carpal tunnel with a reduced nerve diameter of 4mm (Figure 4).

**Follow-up and outcomes:** the evolution was spectacular on the sensory level with the disappearance of paresthesia and pain in the right hand with recovery of the nerve at S4, but on the motor level the motor recovery was rather slow with a rating of M3 at 6 months after the operation.

**Patient perspective:** the patient showed her satisfaction with the neurolysis she underwent. She reported that she can sleep at night. The patient is also satisfied with the scar.

## Discussion

Mucopolysaccharidosis, first described in 1919, is an autosomal recessive lysosomal disease of overload linked to the deficiency of an enzyme of GAG metabolism, IDUA [1]. The estimated incidence of this disease is 1/100,000. This enzyme deficiency induces multisystemic damage due to the accumulation of heparan sulphate and dermatan sulphate. The disease is characterized by the progressive appearance of organomegaly (hepatomegaly and splenomegaly), recurrent inguinal and umbilical hernias, recurrent ENT infections (acute otitis media, sinusitis, etc.), conductive deafness (linked to sero mucosal otitis), enlarged tonsils and adenoids, corneal opacities with decreased visual acuity, characteristic dysmorphia with thickened features, hirsutism, and macroglossia, orthopedic complications with joint stiffening due to multiple dysostoses, risk of spinal cord compression, cardiomyopathy, valvopathies, respiratory insufficiency, motor and cognitive retardation. The phenotype of MPSI is variable in severity depending on the residual enzyme activity, and the severity of the disease. The phenotype of MPSI varies in severity depending on the residual enzyme activity. In the severe form, known as Hurler's disease, patients usually show no clinical signs at birth. During the first months of life, the phenotype appears progressively. The diagnosis is usually made between 4 and 18 months of age in the face of progressive multi-visceral involvement. Symptoms are severe and patients with Hurler disease usually die in the second or third decade if not treated appropriately [2]. There are also milder forms of MPSI without neurocognitive impairment: Scheie's disease. Signs appear after 5 years. The diagnosis is generally made later in the second decade. Inter-mediate forms called Hurler-Scheie have been described. It currently appears that these different clinical pictures constitute a phenotypic continuum from the Hurler form to the Scheie form [3].

Carpal tunnel syndrome (CTS) is a frequent finding in various MPS, including MPS types I, II and VI. Indeed, according to Aldenhoven M *et al*. [4], this syndrome is common in MPS I but rare in children (60% of CTS in children are due to MPS) [5]. Diagnosis is difficult, with patients with mucopolysaccharidosis reportedly diagnosed with carpal tunnel syndrome at a late and irreversible stage [6]. Delayed diagnosis at a motor deficit stage requires early detection of CTS with clinical examination every 6 months and neurophysiological investigation every 12 months. Carpal tunnel syndrome may be associated with protruding fingers, particularly in attenuated phenotypes [7]. Compression of the median nerve within the carpal tunnel in MPS is caused by excessive GAG deposition in the connective tissue of the flexor retinaculum and synovial sheath of the flexor tendons associated with altered skeletal anatomy [8]. Clinically, the classic symptoms of CTS, such as numbness, tingling and night pain, are rarely reported by patients with MPS and these symptoms may be masked by other features of the disease, such as joint stiffness, as well as sensitization tests of the median nerve at the wrist: tinel's and phalen's signs may be absent in children. Therefore, the diagnosis of CTS is often delayed in these patients, explaining the frequent motor involvement with thenar muscle amyotrophy and thumb opposition deficit [9]. Carpal tunnel syndrome may be associated with flexor tenosynovitis, particularly in the attenuated subtypes, caused by GAG deposits in the capsular tissues of the flexor joints or tendons. Electromyography, which is difficult to perform in children, can help in the diagnosis, which remains clinically based. Ultrasound of the median nerve and carpal tunnel is an attractive alternative or adjunct to nerve conduction studies as a screening modality for carpal tunnel syndrome in patients with mucopolysaccharidosis. It is very tolerable in children, with a reported sensitivity of up to 95% [10]. Nerve ultrasound, however, is user-dependent and can be difficult to interpret. Management is surgical, and must be rapid before the motor deficit sets in and is difficult to recover. We perform the classic carpal tunnel approach, allowing sectioning of the thickened flexor retinaculum and release of the median nerve.

## Conclusion

Carpal tunnel syndrome in children is rare. The clinical picture is not very noisy. It is a serious condition as it can lead to significant motor sequelae impinging on the function of the hand (pollici- digital clamp). Early detection of carpal tunnel syndrome in MPS prevents these complications. It is therefore important to be vigilant and to have easy recourse to complementary examinations in a child with a lysosomal overload disease (electromyogram, ultrasound). Early open surgical treatment gives good results and avoids functional sequelae.
